# Etymologia: tularemia

**DOI:** 10.3201/eid1311.070932

**Published:** 2007-11

## [t-lə-rē-mē-ə]

An infectious, plaguelike, zoonotic disease caused by the bacillus *Francisella tularensis*. The agent was named after Tulare County, California, where the agent was first isolated in 1910, and Edward Francis, an Officer of the US Public Health Service, who investigated the disease. Dr. Francis first contracted deer fly fever from a patient he visited in Utah in the early 1900s. He kept a careful record of his 3-month illness and later discovered that a single attack confers permanent immunity. He was exposed to the bacterium for 16 years and even deliberately reinfected himself 4 times.

Tularemia occurs throughout North America, many parts of Europe, the former Soviet Union, the Peoples Republic of China, and Japan, primarily in rabbits, rodents, and humans. The disease is transmitted by the bites of deerflies, fleas, and ticks; by contact with contaminated animals; and by ingestion of contaminated food or water.

Clinical manifestations vary depending on the route of introduction and the virulence of the agent. Most often, an ulcer is exhibited at the site of introduction, together with swelling of the regional lymph nodes and abrupt onset of fever, chills, weakness, headache, backache, and malaise.

